# Supplementary material for: The Use of Point-of-Care Ultrasound (POCUS) in the Diagnosis of Deep Vein Thrombosis
Source: J Clin Med. 2021 Aug 30;10(17):3903. doi: 10.3390/jcm10173903 (PMC8432124; doi:10.3390/jcm10173903)
Supplement: Supplementary file 1 [file jcm-10-03903-s001.zip › Supplementary Videos.pdf]

Supplementary Videos

Supplementary video 1. 3-point POCUS technique for evaluation of acute proximal DVT in the right lower extremity. The “frog-leg” position is depicted. Veins were fully compressible in all points and no DVT was appreciated.
Abbreviations: Rt=right, CFV=common femoral vein, GSV=great saphenous vein, FV=femoral vein, DFV=deep femoral vein

Supplementary video 2. 3-point POCUS technique for evaluation of acute proximal DVT in the left lower extremity. Veins were fully compressible in all points and no DVT was appreciated.
Abbreviations: Lt=left, CFV=common femoral vein, GSV=great saphenous vein, FV=femoral vein, DFV=deep femoral vein

Supplementary video 3. Detection of left femoral vein DVT at the bifurcation. The vein lacks compression.

Supplementary video 4. Detection of left femoral vein DVT at the great saphenous vein – common femoral vein junction. The vein lacks compression.

Supplementary video 5. Detection of right femoral vein DVT at the great saphenous vein – common femoral vein junction. Thrombus is visible.

Supplementary video 6. Detection of left femoral vein DVT distally. Thrombus is visible.

Supplementary video 7. Detection of right popliteal vein DVT. Thrombus is visible.

Supplementary video 8. Detection of left popliteal vein DVT. The vein lacks compression and thrombus is visible.

Supplementary video 9. A Baker's cyst can be mistaken for a non-compressible popliteal vein and lead to misdiagnosis of popliteal thrombosis.

Supplementary video 10. An enlarged inguinal lymph node can be mistaken for thrombus and lead to misdiagnosis of thrombosis.
